# Shared Decision-Making to Improve Health-Related Outcomes for Adults with Stroke Disease

**DOI:** 10.3390/healthcare11121803

**Published:** 2023-06-19

**Authors:** Lidia Bajenaru, Alexandru Sorici, Irina Georgiana Mocanu, Adina Magda Florea, Florina Anca Antochi, Athena Cristina Ribigan

**Affiliations:** 1 Department of Computer Science, Faculty of Automatic Control and Computers, University Politehnica of Bucharest, 313 Splaiul Independentei, 060042 Bucharest, Romania; alexandru.sorici@upb.ro (A.S.); irina.mocanu@upb.ro (I.G.M.); adina.florea@upb.ro (A.M.F.); 2 Department of Neurology, University Emergency Hospital Bucharest, 169 Splaiul Independentei, 050098 Bucharest, Romania; cercetare@suub.ro (F.A.A.); athena.ribigan@umfcd.ro (A.C.R.); 3 Department of Neurology, Faculty of Medicine, University of Medicine and Pharmacy “Carol Davila” Bucharest, 37 Dionisie Lupu, 020021 Bucharest, Romania

**Keywords:** stroke, data collection journey, digital transformation, patient engagement, shared decision-making

## Abstract

Stroke is one of the leading causes of disability and death worldwide, a severe medical condition for which new solutions for prevention, monitoring, and adequate treatment are needed. This paper proposes a SDM framework for the development of innovative and effective solutions based on artificial intelligence in the rehabilitation of stroke patients by empowering patients to make decisions about the use of devices and applications developed in the European project ALAMEDA. To develop a predictive tool for improving disability in stroke patients, key aspects of stroke patient data collection journeys, monitored health parameters, and specific variables covering motor, physical, emotional, cognitive, and sleep status are presented. The proposed SDM model involved the training and consultation of patients, medical staff, carers, and representatives under the name of the Local Community Group. Consultation with LCG members, consists of 11 representative people, physicians, nurses, patients and caregivers, which led to the definition of a methodological framework to investigate the key aspects of monitoring the patient data collection journey for the stroke pilot, and a specific questionnaire to collect stroke patient requirements and preferences. A set of general and specific guidelines specifying the principles by which patients decide to use wearable sensing devices and specific applications resulted from the analysis of the data collected using the questionnaire. The preferences and recommendations collected from LCG members have already been implemented in this stage of ALAMEDA system design and development.

## 1. Introduction

Neurological diseases are the third most common cause of disability and premature death in Europe. Among neurological diseases, Parkinson’s Disease, Multiple Sclerosis and stroke (collectively named PMSS) represent a severe medical condition for which new solutions for prevention, monitoring, and adequate treatment are needed [1,2,3,4]. PMSS neurological diseases are particularly complex, characterized by a wide range of motor and non-motor symptoms [4,5,6].

Stroke is one of the leading causes of disability and death worldwide [7]. Tracking the cognitive and motor recovery of stroke patients is an important activity to ensure that patients can make steady steps toward regaining autonomy and improving their quality of life. Due to a large number of patients with stroke-related motor and neurocognitive deficits, the long-term accessibility of this patient group to rehabilitation centers remains low, even in well-developed countries [8,9]. This complexity, combined with the lack of objective biomarkers of disease progression, poses a challenge in assessing disability and progression. Traditional assessment scales are subjective, episodic, and usually limited to personal visits, and traditional scales are limited and inadequate to capture fluctuations in symptoms and are unable to provide a comprehensive assessment.

Digital technology has enormous potential to transform clinical research and care in neurological diseases [10,11]. Digital therapy involves the use of digital technologies in combination with evidence-based medicine to streamline treatment and personalize patient intervention. This approach helps improve access to evidence-based interventions and provides patients, who have neurological diseases, greater control and agency over their treatment. It is worth noting the need to involve all stakeholders (patients, care partners, healthcare providers, IT professionals and companies) is to ensure that feasible, realistic, and evidence-based solutions are generated [12,13,14,15,16].

For a better understanding of the daily evolution of neurological diseases, the method of empowering patients in making treatment decisions is an innovative and efficient method [17,18]. Current medical decision-making models for brain diseases consider the following aspects: therapeutic strategy; avoiding significant delays before a person with symptoms consults a neurologist for diagnosis and treatment; the need for early and timely intervention; a full range of therapies that can reduce disease activity, thus improving the chances of finding the best option for each person with a brain disease. To achieve these points, regular monitoring of the person’s condition is fundamental and a cornerstone of the strategy to improve the current state of decision making [17].

Shared Decision Making (SDM) is a process by which healthcare professionals and patients collaborate together to choose treatments based on both patient preferences and clinical evidence [18]. The SDM methodology promotes the active involvement of the patient in decisions regarding treatment and personal health care [18,19]. The purpose of applying the shared decision-making principle is to define the implementation of the patient medical journey monitoring process, which results in a customizable Data Collection Journey. The outcome of any therapeutic approach often depends as much on the active involvement of individuals in the decision-making process as it does on the tools and methods used. SDM can help improve patients’ knowledge and understanding of the importance of being involved in decision making, and accounts for patients’ preferences along with the best medical evidence to make the best decision [20]. 

The ALAMEDA project (Bridging the Early Diagnosis and Treatment Gap of Brain Diseases via Smart, Connected, Proactive and Evidence-based Technological Interventions) proposes a monitoring solution for patients with Parkinson’s disease, Multiple Sclerosis, and stroke, using multiple sensors and specific applications to collect information on the condition of health and aspects of lifestyle, activity level, sociability, and mood. The heterogeneous data collected are intelligently integrated, resulting in a comprehensive picture of the patient’s current condition and its evolution over time, allowing caregivers to determine the best care approach in each individual case. The collection of data from patients is either automatic (via wearable devices) or at the patient’s request (questionnaires, user input requirements, etc.). Our SDM process, based on the MULTI-ACT methodology [21], proposes the engagement of patients and caregivers in the co-design of the research and innovation route undertaken in the project. This involves identifying options for each pilot participant’s individual data collection journeys and expressing preferences about those options. Thus, we adapted the original SDM principles [19] to inform the patients about the available customization points of the monitoring process and to decide how their data collection path and data interaction methods will look like.

This paper presents the key elements that have defined the overall framework for the investigation, prevention, and treatment of stroke through the process of Shared Decision Making in the ALAMEDA project.

### Shared Decision-Making for Stroke Disease

Stroke survivors face a long-term chronic condition; rehabilitation is usually started during hospitalization and continues for 3–4 months in a neurorehabilitation unit or at home, usually under the guidance of a specialist. Stroke patients suffer a range of long-term disabilities and complications. Most people with stroke have problems with the upper and lower extremities, most commonly paresis, which is the key impairment in most cases. In addition, movement and balance difficulties are two of the most devastating sequelae of stroke, and restoring gait is often one of the primary goals of rehabilitation, as a high percentage of people report having fallen at least once in the first 6 months after a stroke. In the range of problems related to physical impairment, cognitive and emotional abilities can also be compromised. For example, aphasia is a frequent communication barrier between the physician and the stroke patient, even after the acute phase has passed. and requires formal and informal clinicians and caregivers to seek alternative means of communication. People who suffer a stroke are often affected by pain, but also by anxiety induced by their conditions (for example, fear of loss) or depression induced by memory loss (for example). All the conditions mentioned above are just some of the characteristics of stroke patients.

While stroke treatment has been considerably advanced in recent decades, leading to both mortality and morbidity reduction, improvements in stroke prevention and recovery therapy are needed. Following a stroke, many patients are left with some degree of motor impairment [22]. The enormous burden associated with motor impairments, in terms of economic costs [23] the need for rehabilitation [24], and years of life adjusted for disability [25], require the use of new techniques and methods for improved care that are optimized for acute and chronic strokes. Stroke is a personal experience; every stroke survivor has a certain imprint of this condition [26]. Moreover, many strokes could be prevented by limiting the risks through lifestyle changes and working with the healthcare team. 

Many clinical trials investigate the primary and secondary prevention of stroke, acute treatment, and intense rehabilitation programs [27]. Patients with stroke have identified specific challenges for their recovery, such as returning to routine activities, depression, and emotional issues, which have not been taken into account. The individualization of treatments in recent years has led to an increasing number of precision medicine efforts [28]. The patient’s characteristics, conditions, preferences, and desired results are taken into account for the safety and clinical efficacy of the various treatment options [29]. Specific characteristics of the patient, hospital, and provider may directly affect the outcome of stroke [30].

Predicting the impact of stroke provides particularly important information for planning care and establishing approaches. Modeling approaches are proposed to estimate 30-year projections of stroke epidemiology in the European Union [7,31]. The physicians must know the values and goals of patients and the evidence behind the various diagnostic and treatment options. This is relevant in the context of decision-making during stroke care, rehabilitation strategies, and secondary stroke prevention. [25]. Time and accurate decision making are very important for stroke care, for which several factors have been shown to have an effect on outcomes [32]. The use of SDM in the prevention and treatment of stroke clearly leads to improved diagnostic or therapeutic options, such as efficacy, quality of life, comfort, and cost. They can also lead to better patient information and the choice of key decisions [33]. 

Studies show that although there is a lack of research on SDM in stroke care, decision-making tools are available for certain aspects, such as anticoagulation to prevent stroke in atrial fibrillation [25]. SDM has a decisive role due to the differences between the individual risks based on comorbidities [34]. In a randomized clinical trial of 922 patients with atrial fibrillation (AF) and 115 clinicians, the use of the Anticoagulant Choice Shared Decision-Making encounter tool led to several improvements in decision making, providing support to the clinician without changing the anticoagulant treatment rates. The results indicate that the use of a common decision-making tool in the clinical setting contributes to the care of patients with atrial fibrillation who are considering anticoagulant treatment [35]. In [29] an SDM solution, patient-focused research on stroke outcomes is preferred, and efficacy research (PROSPER) has been developed to help patients, clinicians, and other stakeholders make informed decisions about caring for stroke and improving outcomes by researching patient-centered comparative efficacy. PROSPER aims to improve the decision-making process in the care of stroke and patient outcomes, reflecting the patient’s individual preferences, needs, and values. Time spent in and out of the hospital at “home time” and major adverse cardiovascular events is the main outcome identified and prioritized by stroke patients. Patient engagement has been an important factor for success, in identifying research needs and outcomes that are most desired and prioritized by patients. The research plan was based on a series of focus groups with patients who suffered severe forms of stroke. Data collected from a personalized survey of stroke patients provided information on the most appropriate treatment decisions. The knowledge gained and reviews by stakeholders, turned these identified research needs into patient-centered research questions, which were translated into a study protocol. 

How clinicians make decisions and predict results is based on acquired knowledge, assessment information, and others, using risk-assessment tools or a combination of the above. A better understanding of the decision-making process in acute ischemic stroke may be better if the doctor knows about the sources of error and implements the right decisions when faced with difficult clinical scenarios [36].

Another aspect considered by the professionals in the field is the assessment of rehabilitation, which is essential to determine an appropriate intervention for a patient after a stroke [37]. In work such as [38], this concern for therapists to assess the patient’s functional abilities is addressed, and an intelligent decision-support system is presented that could identify important features of assessment, using reinforcement learning to assess movement quality and summarize patient-specific analysis. Also, a decision-support system that identifies important features to predict the quality of movement and to generate user-specific analyses as explanations of predictions is presented. Appropriate clinical intervention for a patient with musculoskeletal and neurological disorders after a stroke requires a correct evaluation [39]. The use of computer-assisted decision support tools that can monitor and assess chronic diseases, using sensors and machine learning technologies, has proven to be much more effective [40]. Paper [41] presents a series of research that aim to highlight the state of the art, limitations, and concerns, as well as suggestions for elderly care and Kinect-based stroke rehabilitation applications. Kinect applications have the potential to reduce this impracticality through guided interactive rehabilitation and virtualized therapists. 

In the ALAMEDA project, the stroke rehabilitation pilot aims to study the use of a set of metrics based on data collected with wearable devices and on analysis software tools that complement and extend the information obtained through typical processes applied to chronic patients who have suffered an accident cerebrovascular. In order to ensure that stroke patients regain their autonomy and improve their quality of life, it is necessary to follow the cognitive and motor rehabilitation process.

Access to rehabilitation centers for patients with motor and neurocognitive deficits related to stroke is not so easy and within reach. The ALAMEDA project envisages monitoring at certain times during the project, but also extended times, in order to allow doctors to have a continuous update of the patient’s rehabilitation process, between clinical visits.

The stroke pilot assessment will be using the Artificial Intelligence/Machine Learning algorithm to distinguish between different levels of severity for movement and cognitive impairment, as quantified by standard neurological tests.

The above considerations motivate the study protocols and data collection journeys that we detail in the sections that follow.

For the purposes mentioned, in the following sections, we mention the data collection journeys, the SDM process implemented, the results obtained that led to the personalization of the use of the devices and applications developed in the project, and already implemented results.

## 2. Material and Methods

In the ALAMEDA project, the stroke pilot study is a 1-year longitudinal observational study; 15 stroke rehabilitation patients are enrolled in the observational study at the Department of Neurology of the Bucharest University Emergency Hospital.

Stroke patients who are included in the study must meet the inclusion and exclusion criteria established by the stroke team. Among the inclusion criteria, we mention that they can be between 18 and 85 years old, must have digital skills, are able/have the means to return for re-evaluation in the SUUB clinic and be closely monitored during neurological rehabilitation at home. Among the exclusion criteria, we mention: aphasia, complete bilateral blindness, patients whose muscle strength is severely affected by less than 3/5 points on the MRC scale, and patients with severe neurocognitive disorders who scored less than 10 points on a MOCA questionnaire taken before hospital discharge.

One of the goals of the ALAMEDA project is to develop a continuous digital-transformation methodology that involves a continuous process of remote monitoring using wearable devices and mobile applications for patient-reported outcomes and experience referrals. The type of data recorded and the process to be collected are consistent with the particularities of stroke care needs. Precise targets are defined to make the prediction of the collected data.

Patient data collection is performed in several ways: continuously through PRO referrals and the use of a smart watch to obtain information on daily activity and sleep behavior, and through sets of 2-week intensive motor-monitoring periods at certain milestones, where a list of minimally intrusive wearable devices is monitored.

### 2.1. Patient’s Data Collection Journey for the Stroke Pilot

The goal of the stroke pilot is to develop a set of metrics and expand the monitoring capabilities of neurologists for chronic stroke patients using an AI-based software analytics toolkit. The purpose of this extensive monitoring is to allow physicians to have a continuous update on the patient’s recovery process between clinical visits.

The aim of using SDM in the stroke pilot is to improve rehabilitation treatment for stroke patients. Monitoring the cognitive and motor recovery of stroke patients is an important activity for professionals in the field to ensure the increase of autonomy and the improvement of the patients’ quality of life. The monitoring process for the patient’s healthcare journey is part of process of SDM.

**Figure 1:**
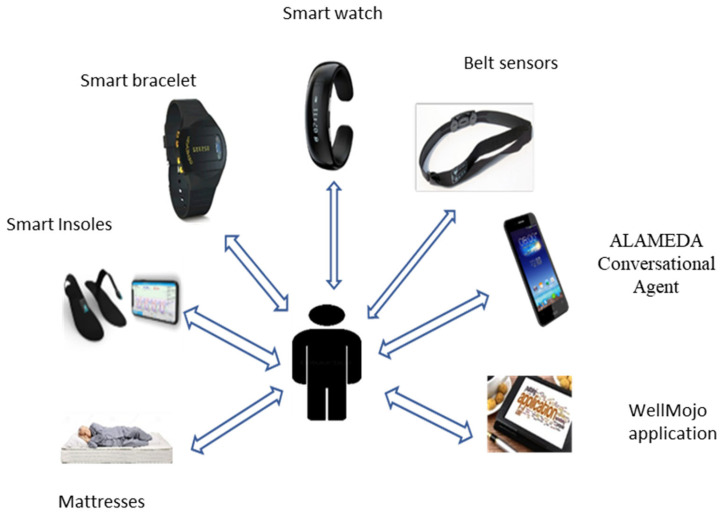
Smart devices and ALAMEDA applications for monitoring patient health parameters.

The Data Collection Journey consists of the patient’s experience in the pilot study regarding the information that will be collected using the devices and software applications established in the ALAMEDA project. The following smart devices and applications are used in our pilot: Smart watch (Fitbit Versa), Smart Insoles (Loadsol-AP insoles developed by Novel Gmbh), Smart Bracelet (ActiveInsights GENEActiv bracelet), Smart Belt (a prototype wearable device developed by a member of the project consortium), a Mattress topper pressure sensor (a prototype device developed by a member of the project consortium), and a suite of mobile applications: ALAMEDA. The Digital Companion (e.g., WellMojo application, ALAMEDA Conversational Agent) allows patients to interact with the ALAMEDA platform to submit Patient Reported Outcomes (PROs), receive notifications and alerts, and monitor their health status across various aspects (Figure 1).

The data collected by these devices and applications are used to better understand the daily evolution of patients when they are enrolled in a rehabilitation process and as an input to a prediction tool for disability improvement using personalized rehabilitation therapies that could be performed at home.

In this regard, variables were identified for continuous passive monitoring, which were discrete for the corresponding end-users involved. The variables cover motor, physical, emotional, and cognitive status, as well as sleep assessment. These will be collected using the mentioned set of wearable devices (Smartwatches, Shoe pressure sensors, smart bracelets, mattresses, belt sensors), and suite of mobile applications ALAMEDA Digital Companion. The variables identified, specific to each disease, were grouped in the predictor (Table 1) and the inferred variables (Table 1). Most predictive variables are data that will be collected using the selected set of wearable devices, as well as through the ALAMEDA digital companion, which facilitates the collection of self-reported patient information. On the other hand, the deduced variables represent neurological tests that will be performed during scheduled clinical visits in the pilot.

The variables are categorized according to the recurrence of their observation. Continuously monitored variables can be monitored over a period of time without interruptions or specific time checks, others may be decided by clinicians as needed. Discretely monitored variables are thus defined according to their nature or the method applied to collect them. In fact, several tests provide a specific time frame for their submission to the patient. The medical partners have established the variables that will be evaluated in the process of monitoring each disease. Many of these are common to all 3 diseases.

All predictor variables are collected using the selected devices and ALAMEDA applications. Variables of type health are collected without explicit patient intervention, using smart devices. Variables of type PRO are collected using explicit questionnaire forms delivered through the ALAMEDA Digital Companion smartphone applications and interactions with the ALAMEDA Conversational Agent, which adapts the PRO content to a conversation form.

The list of predictor variables and inferred variables for stroke grouped on five domains is presented in Table 1.

The patient data collection journey distinguishes between two different data collection modalities, with respect to the distribution of input over time:

- Temporally unbound inputs: data that can be collected from sensors or self-reported by the patient all throughout the duration of the pilot.
- Temporally bound inputs: data that will be collected in a predefined window of time around the milestones defined for the trial (e.g., two weeks before and/or after the milestone).

Data collection can be further characterized by the frequency of the input:

- Continuous input—the patient will provide data by normal wearing of the sensors and consistent response to simple lifestyle and wellbeing questions, with the minimum intrusive level.
- Task-specific input—the patient performs (upon being prompted by ALAMEDA) a specific type of activity at home. The instructions on how to perform the activity have to be available in the ALAMEDA app and the patient can call upon the support of the Patient Engagement Team.

The patient’s data collection journey in stroke considers the following ways of collecting data using established sensors, smart devices, and applications.

**Figure 2:**
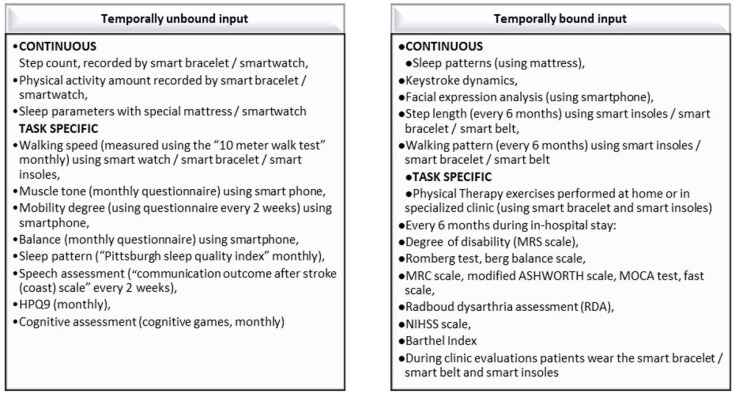
Patient data collection journey specifications for the stroke pilot study.

In Figure 2 are presented Patient data collection journey specifications for the stroke disease pilot study.

### 2.2. Methodology of Shared Decision-Making for Stroke Pilot

The ALAMEDA Shared Decision Model for Brain Disease Assessment draws its development principles from two main sources which are adapted to the objectives of ALAMEDA: the MULTI-ACT Patient Engagement Model [21] and the principles of Shared Decision Making, as understood in the current medical practice. We first present the relevant content of each foundational model and then describe its application to the ALAMEDA pilots.

The objective of the MULTI-ACT project was to develop a strategic Collective Research Governance and Sustainability Model in the area of brain diseases. One of the key aspects of the developed governance methodology is that patients are seen as key stakeholders in the Health Research and Innovation (R&I) processes themselves [21].

The Engagement Coordination Team (ECT) represents mainly primary end users, specifically patients and clinicians was organized. It aims to capture patients’ experiential knowledge and coordinates the involvement of the relevant end users’ community in all operations of setting up and carrying out the ALAMEDA Pilot studies. The ECT furthermore coordinates all training and coaching activities to facilitate the patient engagement. During the execution of the pilot studies, the ECT will provide multi-disciplinary advice to the ALAMEDA consortium on intermediate achievements and suggest corrective actions where needed from the patient perspective.

The ECT for the ALAMEDA Pilot Studies has the following composition:

- Three representatives affected by the neurological conditions under study: one person with Multiple Sclerosis (MS), one person with Parkinson’s Disease (PD) and one person in recovery after a Stroke incident.
- Medical practitioner representatives, each being an expert in one of the three considered neurological conditions.
- One representative from the technical partners of the ALAMEDA consortium.
- One ethics expert.
- One person with expertise in implementing the MULTI-ACT methodology, actively assisting and supporting the activities of the ECT.
- One person acting as the task leader for the stakeholder engagement activities throughout ALAMEDA project.

ALAMEDA envisions that the persons affected by the diseases, whom are members of the ECT, will not only be advisors, but they can also act as ambassadors that engage corresponding communities in order to capture experiential knowledge. Early engagement of patients can lead to greater sustainability through cost reduction, efficiency and efficacy promotion, and would increase the added value of the conducted research. Therefore, patient engagement shall be mission agenda driven and the ALAMEDA consortium identified the R&I steps where patient engagement is a key asset.

#### Local Community Group for Stroke Pilot

To reach out to reference communities in the ALAMEDA stroke pilot-stroke recovery in Romania, the ECT oversees the establishment of Local Community Groups (LCG). The LCGs are the first representation of the larger community that ALAMEDA aims to reach and engage. Apart from the primary end users of the ALAMEDA research, the LCGs extend their composition to include the perspectives of people involved in the care of the primary end users, such as professionals from other healthcare domains (e.g., physical therapists, nurses, psychologists) or the relatives and informal caregivers of the patients. 

The members of the LCG secure the compliance of the R&I activities put in place by ALAMEDA, with the Patient Engagement Guidelines of MULTI-ACT. Concretely, they provide feedback on the proposed usage scenarios and Patient Data Collection Journeys and act as the first testers of the system.

The LCG members are the first to be engaged in these activities. The involvement and relative contribution of the LCG members in ALAMEDA with respect to each of these steps is as follows:

1. Breaking down boundaries: as early testers of the ALAMEDA system, members of the LCG facilitate describe the facilities, infrastructure and research agenda of ALAMEDA to each patient that will be onboarded in the Pilot studies. They would help patients and caregivers understand the monitoring protocol and research questions posed in the project and facilitate end-user engagement in the research activities.
2. Research priorities: LCG members and the patients engage in validation of the usability and personal impact/satisfaction metrics that will be used in the project.
3. Steering institutions: Patients are invited to be members in the Local Community Groups.
4. Design and Plan: LCG members and the patients will participate in customizing the Patient Data Collection Journey, as well as the data interaction modalities (definition of the appropriate means to deliver and receive comprehensive visualizations of the data collected from a person throughout the pilot).
5. Research Execution: LCG members are engaged in the monitoring of ALAMEDA Pilots execution to increase compliance with the monitoring protocol and facilitate meaningful data collection.
6. Evaluation: LCG members and the patients are involved in results assessment relative to the ALAMEDA usability and personal impact metrics, collected through questionnaires that are handed out during Pilot milestones.
7. Translation to the Community: LCG members actively collect feedback on the experience of ALAMEDA pilot study participants and are engaged in the dissemination process of this knowledge to other persons affected by the same neurological disorder.

The aim of applying the principles of Shared Decision Making is to define the implementation of the monitoring process for the patient’s healthcare journey, which leads to a customizable Data Collection Journey. Thus, the general objective of the SDM is to inform the patients about the available customization points of the ALAMEDA monitoring process and to decide what their data collection journey and data interaction methods will look like. We had thus to deal with the customization and adaptation of the original SDM principles to the focus points of the ALAMEDA pilot studies.

**Figure 3:**
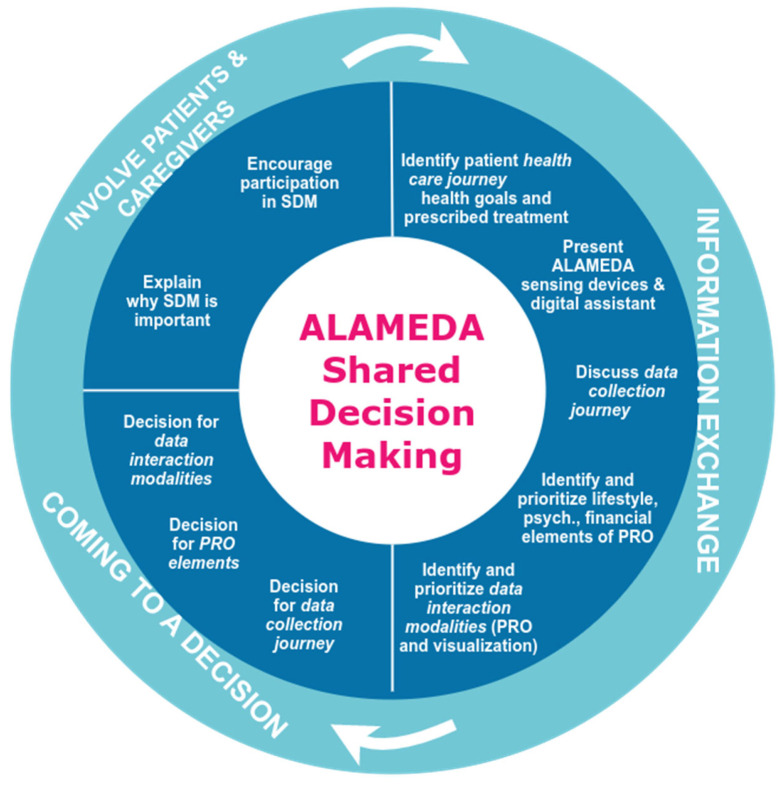
The Shared Decision-Making model applied to ALAMEDA pilot studies.

The process, as applied to the ALAMEDA pilots, is presented in Figure 3.

The Local Community Group for stroke pilot consists of 3 doctors, 1 nurses and 1 physiotherapist, as well as 3 patients and 3 caregivers. All participants were consulted for their opinions and experiences with the aim of helping ALAMEDA researchers to design a monitoring and data collection system that is flexible and adaptable to the specific circumstances and needs of each patient.

LCG for stroke are the initial proxy means by which the ECT engages end-user stakeholders (most notably patients and their caregivers) in the design, customization, prioritizing and evaluation of the ALAMEDA pilot study.

Both the MULTI-ACT guidelines for patient engagement, as well as the general Shared Decision-Making methodology, mention that stakeholder informing is the first step to creating an effective partnership between medical and technical partners, and also the representatives of patient communities.

For debating and collecting information from the LCG members, the MULTI-ACT methodology mentions several methods for public consultation [21], among which are Informed and Deliberative Surveys, as well as Focus Groups. Informed and Deliberative Surveys are a quantitative method by which a preference of people (in terms of single choice, ranking) or their subjective degree of agreement with a statement can be ascertained. Both methods presented above were used to assess the opinions of the end-user representatives and formulate guidelines for the implementation of the ALAMEDA pilot studies.

A Focus Group is a qualitative method used to determine the preferences of people or to evaluate strategies and concepts. Participants are selected according to certain characteristics in common that relate to the research topic and are grouped into 8–10 people. The method is often used to generate or evaluate hypotheses and ideas in conjunction with a quantitative method, or as a primary data-collection method. The Focus Group is a widespread technique of engagement, rooted in qualitative studies and used as a standard way of gathering patients’ input and gaining insight about their views and experiences.

In the context of ALAMEDA LCGs, both methods presented above were used to assess the opinions of the end-user representatives and formulate guidelines for the implementation of the ALAMEDA pilot studies.

The discussions with the LCGs have adopted a two-step approach centered around online meetings (physical meetings were discouraged due to the COVID-19 pandemic) that took place in November 2021. The first round of meetings was an informative one, whereby technical and medical partners explained the objective of the ALAMEDA project and the means (devices, applications and usage methods) by which they will be achieved. The follow-up round involved splitting into focus groups or discussing the results of the questionnaires that had been handed out between the two meetings.

The questions submitted to the LCG members were dependent on the particular health care journey and treatment considerations of stroke disease.

Members of the Local Community Groups (LCG) are the first to undergo the steps presented in Figure 3, such as extracting the shared decision-making guidelines that will be applied to each newly recruited patient in the ALAMEDA pilots.

From the Information Exchange perspective, the discussions in which the LCG members were involved pertain to:

- Identifying the possible health care journeys for patients, i.e., consideration of the most common treatment trajectories taken by persons affected by PMSS and accounting the most common patient concerns.
- A clear presentation of the ALAMEDA sensing devices and software applications (e.g., ALAMEDA digital companion, ALAMEDA conversational agent) and of the research objectives considered by the medical experts.
- A discussion around the when, how and with what of the data collection process during the pilots, as well as the prioritization of patient-reported outcomes (PRO) referring to non-disease related factors (e.g., psychological, social status, quality of life, financial issues).
- Identification and prioritization of options for data interaction (i.e., the technical means by which participants in the pilots submit and visualize their data, respond to notifications or engage in dialog with the ALAMEDA Conversational Agent).

The general objective of the ALAMEDA SDM is to inform the patients about the available customization points of the ALAMEDA monitoring process and to arrive at a decision of what their data collection journey and data interaction methods will look like within the ALAMEDA pilots. It is, therefore, the customization and adaptation of the original SDM principles to the focus points of the ALAMEDA pilot studies, which are observational rather than interventional in nature.

The guidelines specify principles by which clinicians and patients decide to employ wearable sensing devices, perform patient-reported data collection, and use the digital companion and/or conversational agent applications developed within ALAMEDA. 

To define the proposal guidelines, the MULTI-ACT methodology is followed, whereby patients and caregivers are engaged in the co-design of the Research and Innovation path undertaken by the ALAMEDA project. Among other things, this involves identifying the options in the individual Data Collection Journeys of each pilot participant and expressing the preferences with respect to these options.

The questions submitted to the LCG members were dependent on the particular data collection journey and treatment considerations of stroke. However, with respect to the organization of the ALAMEDA pilot studies, the following key issues are presented in Table 2.

During the meetings held with the members of the Local Community Group, they were informed about the purpose of the ALAMEDA project, the tasks that lie ahead for each patient involved, and also the wearable devices that will be used. 

Each member of the Local Community Group had to fill in a questionnaire consisting of 13 questions (Table 3). After gathering their answers, the LCG members raised a number of issues and comments that were discussed.

### 2.3. The Specific Questionnaire Result of Local Community Group consultation

The specific questionnaire used to get patient input on the data collection journey included 13 defined questions classified into 5 sections, as presented in Table 3:

To simplify the data collection process, it was chosen to translate the questionnaire into a digital format and present it using smart devices such as tablets or phones.

The answers to the questions are different, either by choosing some variants from those mentioned or by using a five-point Likert scale (1 = strongly disagree to 5 = strongly agree/1 = not at all to 5 = very).

The results were carefully analyzed by the technical partner University Politehnica of Bucharest.

## 3. Results Obtained by Consulting the Members of the LCG

### 3.1. Preferences on Device Wearing 

The personalization of the Stroke data collection journey results from the interpretation and application of the stroke patient’s preferences according to the presented methodology are discussed below. 

According to the majority of respondents, 45.5% “At home” is the is the preference for carrying out specific devices during home exercises and in equal number of cases 27% prefer to either wear the devices during physical therapy sessions, either to have the possibility of deciding between the existing options (Table 4).

With regard to question 2 “If a patient undergoes physical therapy, is he/she willing to wear the devices during those sessions?”, all respondents agreed.

### 3.2. Preferences for Filling out PROs

Most of the respondents, 54.5%, prefer to answer the questionnaire using a standard digital interface, while 36.4% want to be able to choose between the available options (Table 5).

### 3.3. The Preferences for Using the Conversational Agent

Most of the patients (over 63%) prefer to use the conversational agent for a quick query for own health status; 45.5% of the patients are willing to use the conversational agent to report on their psychological, societal or financial situation, and 90.9% of the patients consider that the ALAMEDA conversational agent can be used to collect data about non-disease related factors from the caregivers (Table 6).

### 3.4. Preferences Regarding the Disease Self-Management Module

The existence of Disease Self-Management within ALAMEDA is considered very necessary by 5 participants, and the method preferred for the disease self-management module is to visualize their own data on a smartphone or by watching video tutorials on the ALAMEDA app, in which a medical expert explains disease management techniques by the same number of respondents (Table 7).

### 3.5. Preferences and Other Aspects

The collection of location data should only be performed on request and at a general level of granularity (not at GPS detail resolution), 6 participants considered.

The following takeaways are relevant with respect to establishing the ALAMEDA guidelines for the stroke pilot:

- The specific devices (smart bracelets, smart insoles, smart belts) will be worn by all patients during home exercises. The devices must be worn by those patients that undergo physical rehabilitation sessions. The patients need to use the ALAMEDA application to label the type, start and end time of physical rehabilitation exercises (according to a predefined list of exercises).
- There were predominant preferences for standard interfaces to fill out medical questionnaires and also predominant preferences for stage-wise fill out of the questionnaires. The majority of LCG members agreed that the conversational agent should be maintained as an option to fill in medical questionnaires.
- The Conversational Agent acts as a rapid interface and it is used to report on non-disease-related aspects (taking into account the records of each patient’s emotional status). The Conversational Agent interface is also accepted to be used to receive input from the caregivers.
- The ALAMEDA application must support the quick visualization of patient-submitted information and video tutorials from medical experts.
- Localization data collection must be made only upon request and at a general level of granularity. The smartphone on which to run the ALAMEDA application (own phone or one provided within the project) is a choice that will be left as a personal preference for each participant that enters the study.

#### Discussion Related to Technical Aspects of Wearable Devices and ALAMEDA Applications

After preliminary testing of the wearable devices, as well as the ALAMEDA applications by patients and medical professionals, the following assessments were collected.

Feedback (from doctors):
  - Applications to collect questionnaires were easy to use but a better interaction in natural langue would be desirable.
  - Insoles. The following problems for the patients wearing insoles were detected:
    − Attachment to the hospital slippers-patients is not allowed with other type of shoes, it is difficult to wear Snickers during hospitalization;
    − Turn off due to the detachment of the plastic piece that holds the battery
    − The smartphone supporting the insole application should be kept close to the patient, otherwise the insoles will disconnect
    − Some patients remove the insoles them because they seem not to be properly attached and they fear they might fall
  - Belt. The sending data indicator of the belt was not always functioning.
    − Mini smart-mattress. Smart-mattress must be restarted with a pin after every patient–impossible to configure it without restarting
Feedback (from patients):
  - Applications to collect questionnaires were easy to use but a better interaction in natural langue would be desirable.
  - Insoles:
    − Difficult to wear in slippers
    − Most of the patients had the tendency to fall: they slipped when wearing the insoles
    − They prefer using them only during the exercises
    − Difficulties in changing the batteries of the insoles: almost always changed by a team member
  - Belt: Difficult to wear due to weight. They preferred using it during exercises
  - Mini smart-mattress: No problem except the fact that one patient tried to install the mattress at home but he could not manage it.

The mentioned aspects were taken into account leading to the improvement of ALAMEDA technology.

### 3.6. Guidelines for Stroke Pilot

The results from the specific questionnaire and focus group discussions are synthesized into a set of guidelines that cover the following general and pilot-specific aspects:

- General Guidelines: Inform the patient and caregiver about project goals, devices, and applications used in the project, and discuss the data collection journey and data viewing options
- Pilot Specific Guidelines: discussion over data collection journey for specific devices (smart belt, smart bracelet, and smart insoles), and over content and the method of transmitting the patient-reported outcomes related to impact on activities of daily living

### 3.7. Specific Guidelines for Stroke Pilot

#### 3.7.1. Discussion over Data Collection Journey for Specific Devices

##### Participants: Patients, Clinicians, Physical Therapists, Technical Support

The data collection journey for stroke patients varies depending on the severity of the impact on mobility and cognition.

Clinicians and patients discuss the treatment plan and the ability (in terms of time availability and financial aspects) of the patient to undergo physical therapy sessions either at home or in a specialized care facility.

For patients who want/need to undergo such a treatment, clinicians and physical therapists present the option to wear the specific devices (smart belt, smart bracelet, smart insoles) during the physical therapy sessions. They explain the benefits in terms of additional data which allows for the evaluation of patient progress/regress over time in performing the rehabilitation exercise.

Clinicians and physical therapists describe to the patient the list of exercises that can be annotated in the ALAMEDA Digital Companion application. Technical Support members provide a demonstration of the annotation process.

Clinicians take note of the patient decision. For patients who agree, clinicians amend the stroke Data Collection Journey to issue the specialized devices earlier than the required two weeks before clinical evaluation milestones.

#### 3.7.2. Discussion over Content and Method of Transmitting PROs Related to Impact on Activities of Daily Living

##### Participants: Patients, Clinicians, and Technical Support

Under the Quality of Life and Activities of Daily Living category of the stroke Data Collection Journey, Patients fill in the ACTIVLIM questionnaire [42] about the difficulty to perform Activities of Daily Living on a weekly basis.

Patients and clinicians discuss selecting the most appropriate list of questions from the questionnaire, corresponding to the activities of daily living that are most commonly executed by the patient. Technical Support members take note of the patient selection to enable it in the questionnaire interfaces (ALAMEDA Digital Companion or ALAMEDA Conversational Agent).

The patient and clinician discuss the schedule for administering the ACTIVLIM questionnaire; the options are:

- Fill in the entire questionnaire upon each received notification.
- Notifications display only two to three questions per day, such that the full questionnaire is completed over the course of a week.

Technical Support members take note of the patient’s decision to configure the notification service in the ALAMEDA Digital Companion for this Patient.

##### Opportunities, Disadvantages and Barriers of SDM Model

Stroke disease, a multifaceted condition that can include a range of motor and non-motor symptoms, requires an integrated, interdisciplinary approach to treatment.

Involving stroke pilot patients in the decision-making process may lead to an improved knowledge/understanding of the options available in treatment/investigation, including the potential benefits and risks of each option, and increased satisfaction and trust in the healthcare team.

Part of SDM process is collecting data specific to the stroke pilot, respectively, for monitoring the patient’s healthcare journey. Using digital technology, smartphones and wearable devices proposed in the ALAMEDA project, it may possible to evaluate, objectively, frequently and remotely, the different facets of movement disorders associated with neurological diseases in a natural environment. Also, the evolution of the disease can be identified early, facilitating the identification of treatments that modify the disease. This opens up new perspectives on disease progression that complement standard clinical assessments and allow for a profound clinical phenotyping of neurological diseases [43]. Certainly, digital instruments have enormous potential to improve care, research, and outcomes in stroke disorders. Data collected from wearable sensors, and specific applications, would be synchronized with other data sources (e.g., activity tracking tools, sleep tracking tools, tremor) that could be passively collected during everyday life. It could lead to patients completing short periodic assessments of non-motor symptoms (e.g., anxiety, mood, cognition) and motor status (gait, balance), which could be integrated with data on when medications were taken. Relevant data would be shared and analyzed to allow for personalized digital interventions. 

However, barriers identified in previous research [43,44], such as those from doctors, patients, family, culture and system [45], lack of familiarity and experience with SDM [46], use of medical terminology, lack of continuity of care, doctor’s knowledge of evidence, doctor–doctor–patient relationship, insufficient explanations, the ability of the patient and families to understand and use health information [47], the lack of resources and time [48], can also impact our research. Strategies for overcoming these barriers and facilitating SDM include clinician motivation, patient participation, appropriate timing, and process support tools, such as decision aids. 

The most commonly described facilitators of SDM are related to the clinician’s motivation and perception that SDM improves the clinical process and patient outcomes [46]. Patient-identified facilitators refer to issues such as continuity of care, good patient-physician relationships, trust, adequate time, involvement of various members of the healthcare team, encouraging patients to participate and ask questions, providing sufficient information, using simple language, and patient involvement in the process [49].

## 4. Conclusions

Stroke is a disabling disease that leads to many challenges in terms of disease progression, progression, and severity for patients, physicians, and caregivers.

This research presents the methodological framework for investigating key issues for personalizing the stroke pilot data collection journey.

The SDM model proposed in the stroke pilot contributes to making personalized medical decisions related to goals of care, diagnostic evaluations, rehabilitation strategies, or secondary prevention of stroke, using the best medical evidence. The added value of the SDM model consists of the personalized application of the guidelines of the validated MULTI-ACT methodology, which proposes the involvement of patients and caregivers in the co-design of the research and innovation route undertaken in the project.

The result of consultation with members of the Local Community Group and the application of a specific questionnaire is a set of preliminary guidelines that provide options for customizing the data collection journey of patients. The guidelines specify the principles by which clinicians and patients decide to use wearable sensing devices, conduct patient-reported data collection, and use companion digital applications and/or conversational agents developed within ALAMEDA. A follow up is to integrate our SDM model into the AI-based software analytics toolkit. The preferences and recommendations of the LCG members have already been implemented at this stage of the design and development of the ALAMEDA system.

The limitation of this study could be the lack of a significant number of respondents, in order to be able to define a final guide. However, the preferences and recommendations of LCG members were very helpful in the ALAMEDA system design and development process. In this research, the first phase of SDM of stroke patients is presented, in which their preferences and requirements were collected in relation to the type of monitoring and, respectively, the way of using the devices and applications involved in this process. The results obtained from 11 LCG respondents led to the personalization of the initially proposed methodology, by adapting the type of monitoring with wearable devices, the applications developed in the project, and the entire process of evaluating the health condition. Even if the number of respondents is small, due to the effective and very elaborate training process, along with the debates that took place in the LCG team at the consortium level, it was considered to be a very productive and a really fruitful process in improving the components of the ALAMEDA system. 

Next is the second phase of the integration of the proposed SDM model in the process of monitoring the patients proposed in the study, the results of which will be presented later. Also, we aim to apply our study methodology to a larger number of participants, healthcare professionals, patients, and caregivers to obtain a complete set of guidelines and integrate our SDM model into the AI-based software analysis toolkit.

## Figures and Tables

**Table 1:** List of predictor variables and inferred variables for stroke.

Domains	Predictor Variables	Inferred Variables
Domain I Mobility, general motor or physical function	walking pattern, using smart watch/smart bracelet/smart insoles/smart belt, six-meter walk test (6MWT), Romberg’s test, Berg Balance Scale, MRC (Medical Research Council) scale for motor weakness, Modified Asworth scale	step count, recorded by smart bracelet/smartwatch, periods of relative immobility, physical activity amount, recorded by smart bracelet/smartwatch, walking pattern, using smart watch/smart bracelet/smart insoles/smart belt, six-meter walk test 6MWT, ACTIVLIM questionnaire, Dizziness and Balance Questionnaire, Self-assessed questionnaire for muscle tone
Domain II Sleep disorders	Pitsburg Sleep Quality Index (PSQI)	sleep pattern, using mattress/smartwatch, Pitsburg Sleep Quality Index (PSQI), intense sleep monitoring
Domain III Mental and cognitive ability	Montreal Cognitive Assessment (MoCA), Functional Assessment Staging Tool (FAST)	Line tracking test, Virtual supermarket, Keystroke dynamics
Domain IV Emotional status	Patient Health Questionnaire (PHQ9)	Facial expression analysis, Communication Outcome after Stroke (COAST), Patient Health Questionnaire (PHQ9)
Domain V Quality of life and daily living	Radbound Dysarthria Assessment, the National Institutes of Health Stroke Scale (HIHSS), Modified Rankin Scale (MRS), Barthel Index	Modified Fatigue Impact Scale (MFIS), Food Habits Questionnaire (FH-Q)

**Table 2:** The methodological framework for the investigation of the key issues regarding the consultation of LCGs members.

Customization Points/Discussion and Decision Points	Key Issues	Features	Proposed Options
1. Customization points	1.1. Data Collection Journey for specific devices (smart bracelet, smart insoles, smart belt)	Patients are required to express their preferences concerning the time period and place for performing task specific/temporally limited activities which require the use of specific devices	At home, upon notification from the ALAMEDA Digital Companion During physical therapy sessions During the visit to the family doctor Outdoors, during a period of intense walking
	1.2 Submission of Patient Reported Outcomes (PRO)	Preference on the mode of submitting data for each type of questionnaire	Using only standard questionnaire interface Using only conversational agent Using a combination of the conversational agent and standard questionnaires
		Preference on the frequency of reminders to complete/fill a questionnaire	Remind continuously until fulfillment Reminder only in the morning or only in the evening-if no answer, skip request Reminder as invitation from conversational agent to start a dialogue
		Preference for the process of filling out the questionnaires	Always fill in the full extent of the questionnaire Have the ability to Pause/Resume questionnaire during a day/week Have the ability to distribute a questionnaire-based conversation with the conversational agent throughout the day/week
	1.3. Preferred Use of the Conversational Agent	Preference to use only the conversational agent as the interface to submit data from PRO questionnaires Preference to use only the conversational agent for querying about personal health status (as collected by wearables) Preferences to use conversational agent for both input and query	
	1.4. Preference for Visualization of own health status	Through graphs and charts Through table-like history By asking the Conversational Agent	
2. Discussion and Decision points	2.1. Discussion over the functionality and form of the Disease Self-Management Module (means and methods by which a patient can ascertain his/her own treatment progress)	Discussion over whether simple data visualization is sufficient Discussion over use of type and purpose of video tutorials developed by medical professionals Discussion over use of social media groups for patient experience exchange	
	2.2. Discussion over submission of non-disease related factors	Discussion over willingness of patients and caregivers to report on financial situation, social status (employment, work ability, social connections) and psychological factors Discussion over preference of patients to use a questionnaire or the conversational agent to report data on non-disease related factors Decision over use of the conversational agent to collect non-disease related factors from the caregiver (e.g., stress or burden of caregiver)	
	2.3. Discussion over using own smart phone or one given by ALAMEDA to run the Digital Companion and Conversational Agent applications		
	2.4. Discussion over need and permission to collect patient location information during the pilot studies		

**Table 3:** Items in the Questionnaire are discussing the Data Collection Journey customization options within the LCG.

Sections	Questions	Options
1. Preference for wearing the device	1. Where will patients perform specific activities/limited duration activities?	(a) At home; (b) During physical therapy sessions; (c) During a visit to the family doctor; (d) The patient must be able to decide between (a), (b) and (c)
	2. If a patient undergoes physical therapy, is he/she willing to wear the devices during those sessions?	Yes/No
2. Preference for filling out PROs	3. How do patients prefer to answer to a questionnaire?	(a) Standard digital interface; (b) Using the conversational agent; (c) Using a combination of (a) and (b); (d) The patient must be able to choose between (a), (b) and (c)
	4. What is the patient’s preference for receiving notifications to fill out a questionnaire?	(a) App sends notification at any time of day, repeatedly, until the patient fills in the questionnaire; (b) can receive notification only in the morning or in the evening; (c) receives a notification as an invitation to chat with the conversational agent; (d) must be able to choose between (a), (b) and (c)
	5. How do patients prefer to fill in a questionnaire?	(a) always filled in completely (b) filled in partially and completed later in the day/next day (c) filled in as part of a conversation with the ALAMEDA conversation agent (d) the patient must be able to choose between (a), (b) and (c)
3. Preference for using the Conversational Agent	6. What is the preferred use of the conversational agent?	(a) means to send PRO data (b) means to quickie query one’s own health status (c) both (a) and (b)
	7. Do you think that patients and caregivers are willing to use the conversational agent to report on their psychological, societal or financial situation?)	1—strongly disagree, 5—strongly agree
	8. How do patients prefer to offer data unrelated to the disease?	(a) using a standard questionnaire (b) through conversations (c) the patient must be able to choose between a) and b)
	9. Can the ALAMEDA conversational agent be used to collect data about non-disease related factors from the caregivers?	Yes/No
4. Preference regarding the Disease Self-Management Module	10. How necessary is the existence of Disease Self-Management within ALAMEDA?	1—not at all, 5—very
	11. Which method is preferred for the Disease Self-Management module?	(a) on smartphone (b) external discussion group on social media (e.g., WhatsApp group, Facebook group) (c) Video tutorials on ALAMEDA app in which a medical expert explains disease management techniques
5. Preference regarding other Aspects	12. What is the preference of patients in terms of installing the ALAMEDA app on their own smartphone vs. receiving a new smartphone for the duration of the study?	(a) install on own phone (b) receive a new smartphone with a dual SIM card
	13. What is patient preference in terms of location tracking throughout the pilot?	(a) yes, but only if distinction is between: home, work a and other (b) Yes, but only when I want and only by answering to a question, NOT by GPS tracking (c) No, in no way

**Table 4:** Section 1. Preference for wearing the device.

1. Where will patients perform specific activities/limited duration activitie	(a) At home	(b) During physical therapy sessions	(c) During a visit to the family doctor	(d) The patient must be able to decide between (a), (b) and (c)
Nr. Respondents	5	3		3

**Table 5:** Section 2. Preference for filling out PROs.

3. How do patients prefer to answer to a questionnaire?	(a) Standard digital interface	(b) Using the conversational agent;	(c) Using a combination of (a) and (b);data	(d) The patient must be able to choose between (a), (b) and (c) data
Nr. Respondents	6		1	4
4. What is the patient’s preference for receiving notifications to fill out a questionnaire?	(a) App sends notification at any time of day, repeatedly, until the patient fills in the questionnaire;	(b) can receive notification only in the morning or in the evening	(c) receives a notification as an invitation to chat with the conversational agent;	(d) must be able to choose between (a), (b) and (c)
Nr. Respondents	4	2		5
5. How do patients prefer to fill in a questionnaire?	(a) always filled in completely	(b) filled in partially and completed later in the day/next day	(c) filled in as part of a conversation with the ALAMEDA conversation agent	(d) the patient must be able to choose between (a), (b) and (c)
Nr. Respondents	1	5	2	3

**Table 6:** Section 3. Preference for using the Conversational Agent.

6. What is the preferred use of the conversational agent?	(a) means to send PRO data	(b) means to quickie query one’s own health status	(c) both (a) and (b)
Nr. Respondents	1	7	3
7. Do you think that patients and caregivers are willing to use the conversational agent to report on their psychological, societal or financial situation?	1–4	5—strongly agree	
Nr. Respondents	6	5	
8. How do patients prefer to offer data unrelated to the disease?	(a) using a standard questionnaire	(b) through conversations	(c) the patient must be able to choose between (a) and (b)
Nr. Respondents	5	4	2
9. Can the ALAMEDA conversational agent be used to collect data about non-disease related factors from the caregivers?	Yes	No	
Nr. Respondents	10	1	

**Table 7:** Section 4. Preference regarding the Disease Self-Management Module.

10. How necessary is the existence of Disease Self-Management within ALAMEDA?	1–4	5—very necessary	
Nr. Respondents	5	6	
11. Which method is preferred for the Disease Self-Management module?	(a) on smartphone	(b) external discussion group on social media	(c) Video tutorials on ALAMEDA app
Nr. Respondents	5	3	3

## Data Availability

The dataset used and analysed during the current study is available from the corresponding author on reasonable request.

## Abbreviations

MS	Multiple Sclerosis
PD	Parkinson’s Disease
PMSS	Parkinson’s, MS and Stroke
PRO	Results reported by the patient
LCG	Local Community Group
6MWT	Six Meter Walk Test
MRC	Medical Research Council scale for motor weakness
MoCA	Montreal Cognitive Assessment
FAST	Functional Assessment Staging Tool
PHQ9	Patient Health Questionnaire
PSQI	Pittsburgh Sleep Quality Index
HIHSS	National Institutes of Health Stroke Scale
MRS	Modified Rankin Scale
COAST	Communication Outcome after Stroke
MFIS	Modified Fatigue Impact Scale
FH-Q	Food Habits Questionnaire

## References

[1] Borumandnia N., Majd H.A., Doosti H., Olazadeh K. (2022). The trend analysis of neurological disorders as major causes of death and disability according to human development, 1990–2019. Environ. Sci. Pollut. Res..

[2] GBD 2017 US Neurological Disorders Collaborators (2020). Burden of neurological disorders across the US from 1990-2017: A global burden of disease study. JAMA Neurol..

[3] Deuschl G., Beghi E., Fazekas F., Varga T., Christoforidi K.A., Sipido E., Bassetti C.L., Vos T., Feigin V.L. (2020). The burden of neurological diseases in Europe: An analysis for the Global Burden of Disease Study 2017. Lancet Public Health.

[4] Armstrong M.J., Okun M.S. (2020). Diagnosis and Treatment of Parkinson Disease: A Review. JAMA.

[5] Huang J., Chen W., Zhang X. (2017). Multiple sclerosis: Pathology, diagnosis and treatments. Exp. Ther. Med..

[6] Benjamin E.J., Blaha M.J., Chiuve S.E., Cushman M., Das S.R., Deo R., de Ferranti S.D., Floyd J., Fornage M., Gillespie C. (2017). American Heart Association Statistics Committee and stroke Statistics Subcommittee. Heart Disease and stroke Statistics-2017 Update: A Report From the American Heart Association. Circulation.

[7] Wafa H.A., Wolfe C., Emmett E., Roth G.A., Johnson C.O., Wang Y. (2020). Burden of stroke in Europe: Thirty-Year Projections of Incidence, Prevalence, Deaths, and Disability-Adjusted Life Years. Stroke.

[8] Dobkin B.H., Dorsch A. (2013). New evidence for therapies in stroke rehabilitation. Curr. Atheroscler. Rep..

[9] Canova C., Danieli S., Barbiellini Amidei C., Simonato L., Di Domenicantonio R., Cappai G., Bargagli A.M. (2019). A Systematic Review of Case-Identification Algorithms Based on Italian Healthcare Administrative Databases for Three Relevant Diseases of the Nervous System: Parkinson’s Disease, Multiple Sclerosis, and Epilepsy. Epidemiol. Prev..

[10] Thimbleby H. (2013). Technology and the Future of Healthcare. J. Public Health Res..

[11] Kuriakose D., Xiao Z. (2020). Pathophysiology and Treatment of stroke: Present Status and Future Perspectives. Int. J. Mol. Sci..

[12] Ellis T.D., Earhart G.M. (2021). Digital Therapeutics in Parkinson’s Disease: Practical Applications and Future Potential. J. Park. Dis..

[13] Mehl G., Tamrat T., Labrique A., Orton M., Baker E., Blaschke S., BonTempo J., DeBorma N., Eskandar H., Falzon D. (2018). Classification of Digital Health Interventions v 1.0.

[14] Adams J.L., Lizarraga K., Waddell E.M., Myers T.L., Jensen-Roberts S., Modica J.S., Schneider R.B. (2021). Digital Technology in Movement Disorders: Updates, Applications, and Challenges. Curr. Neurol. Neurosci. Rep..

[15] Taylor P. Smartphone Subscriptions Worldwide 2016–2027. https://www.statista.com/statistics/330695/number-of-smartphone-users-worldwide/.

[16] Coravos A., Goldsack J.C., Karlin D.R., Nebeker C., Perakslis E., Zimmerman N., Erb M.K. (2019). Digital medicine: A primer on measurement. Digit Biomark.

[17] Metz M.J., Veerbeek M.A., Twisk J., van der Feltz-Cornelis C.M., de Beurs E., Beekman A. (2019). Shared decision-making in mental health care using routine outcome monitoring: Results of a cluster randomised-controlled trial. Soc. Psychiatry Psychiatr. Epidemiol..

[18] Aoki Y. (2020). Shared decision making for adults with severe mental illness: A concept analysis. Jpn. J. Nurs. Sci. JJNS.

[19] Curtis L.C., Wells S.M., Penney D.J., Ghose S.S., Mistler L.A., Mahone I.H., Delphin-Rittmon M., del Vecchio P., Lesko S. (2010). Pushing the envelope: Shared decision making in mental health. Psychiatr. Rehabil. J..

[20] Armstrong M.J. (2017). Shared decision-making in stroke: An evolving approach to improved patient care. Stroke Vasc. Neurol..

[21] Zaratin P., Bertorello D., Guglielmino R., Devigili D., Brichetto G., Tageo V., Dati G., Kramer S., Battaglia M.A., Luca M. (2022). The MULTI-ACT model: The path forward for participatory and anticipatory governance in health research and care. Health Res. Policy Syst..

[22] Langhorne P., Coupar F., Pollock A. (2009). Motor recovery after stroke: A systematic review. Lancet Neurol..

[23] Luengo-Fernandez R., Violato M., Candio P., Leal J. (2020). Economic burden of stroke across Europe: A population-based cost analysis. Eur. Stroke J..

[24] Cieza A., Causey K., Kamenov K., Hanson S.W., Chatterji S., Vos T. (2020). Global estimates of the need for rehabilitation based on the Global Burden of Disease study 2019: A systematic analysis for the Global Burden of Disease Study 2019. Lancet.

[25] Feigin V.L., Abajobir A.A., Abate K.H., Abd-Allah F., Abdulle A.M., Ferede Abera S., Abyu G.Y., Ahmed M.B., Aichour A.N., Aichour I. (2017). Global, regional, and national burden of neurological disorders during 1990–2015: A systematic analysis for the Global Burden of Disease Study 2015. Lancet Neurol..

[26] Feigin V.L., Nguyen G., Cercy K., Johnson C.O., Alam T., Parmar P.G., Abajobir A.A., Abate K.H., Abd-Allah F., Abejie A.N. (2018). Global, regional, and country-specific lifetime risks of stroke, 1990–2016. N. Engl. J. Med..

[27] Stinear C.M., Lang C.E., Zeiler S., Byblow W.D. (2020). Advances and challenges in stroke rehabilitation. Lancet Neurol..

[28] National Research Council (US) Committee on A Framework for Developing a New Taxonomy of Disease (2011). Toward Precision Medicine: Building a Knowledge Network for Biomedical Research and a New Taxonomy of Disease.

[29] Xian Y., O’Brien E.C., Fonarow G.C., Olson D.M., Schwamm L.H., Hannah D., Lindholm B., Maisch L., Lytle B.L., Greiner M.A. (2015). Patient-Centered Research into Outcomes stroke Patients Prefer and Effectiveness Research: Implementing the patient-driven research paradigm to aid decision making in stroke care. Am. Heart J..

[30] Saposnik G., Johnston S.C. (2014). Decision making in acute stroke care: Learning from neuroeconomics, neuromarketing, and poker players. Stroke.

[31] Shay L.A., Lafata J.E. (2015). Where is the evidence? A systematic review of shared decision making and patient outcomes. Med. Decis. Mak. Int. J. Soc. Med. Decis. Mak..

[32] Fonarow G.C., Smith E.E., Saver J.L., Reeves M.J., Bhatt D.L., Grau- Sepulveda M.V., Olson D.M., Hernandez A.F., Peterson E.D., Schwamm L.H. (2011). Timeliness of tissue-type plasminogen activator therapy in acute ischemic stroke: Patient characteristics, hospital factors, and outcomes associated with door-to-needle times within 60 minutes. Circulation.

[33] Armstrong M.J., Mullins C.D. (2017). Value assessment at the point of care: Incorporating patient values throughout care delivery and a draft taxonomy of patient values. Value Health.

[34] Ferguson C., Hendriks J. (2017). Partnering with patients in shared decision-making for stroke prevention in atrial fibrillation. Eur. J. Cardiovasc. Nurs..

[35] Kunneman M., Branda M.E., Hargraves I.G., Sivly A.L., Lee A.T., Gorr H., Burnett B., Suzuki T., Jackson E.A., Hess E. (2020). Shared Decision Making for Atrial Fibrillation (SDM4AFib) Trial Investigators. Assessment of Shared Decision-making for stroke Prevention in Patients With Atrial Fibrillation: A Randomized Clinical Trial. JAMA Intern. Med..

[36] De Boer M.E., Depla M., Wojtkowiak J., Visser M.C., Widdershoven G.A., Francke A.L., Hertogh C.M. (2015). Life-and-death decision-making in the acute phase after a severe stroke: Interviews with relatives. Palliat. Med..

[37] Webster D., Celik O. (2014). Systematic review of Kinect applications in elderly care and stroke rehabilitation. J. Neuroeng. Rehabil..

[38] Lee M.H., Siewiorek D.P., Smailagic A., Bernardino A., Badia S.B. Learning to assess the quality of stroke rehabilitation exercises. Proceedings of the 24th International Conference on Intelligent User Interfaces.

[39] Lang C.E., Bland M.D., Bailey R.R., Schaefer S.Y., Birkenmeier R.L. (2013). Assessment of upper extremity impairment, function, and activity after stroke: Foundations for clinical decision making. J Hand Ther..

[40] Berner E.S. (2007). Clinical Decision Support Systems.

[41] Adomavičienė A., Daunoravičienė K., Kubilius R., Varžaitytė L., Raistenskis J. (2019). Influence of New Technologies on Post-Stroke Rehabilitation: A Comparison of Armeo Spring to the Kinect System. Medicina (Kaunas).

[42] Batcho C.S., Tennant A., Thonnard J.L. (2012). ACTIVLIM-Stroke: A crosscultural Rasch-built scale of activity limitations in patients with stroke. Stroke.

[43] Dorsey E.R., Glidden A.M., Holloway M.R., Birbeck G.L., Schwamm L.H. (2018). Teleneurology and mobile technologies: The future of neurological care. Nat. Rev. Neurol..

[44] Espay A.J., Hausdorff J.M., Sa’nchez-Ferro A., Klucken J., Merola A., Bonato P., Paul S.S., Horak F.B., Vizcarra J.A., Mestre T.A. (2019). A roadmap for implementation of patient-centered digital outcome measures in Parkinson’s disease obtained using mobile health technologies. Mov. Disord..

[45] Joseph-Williams N., Elwyn G., Edwards A. (2014). Knowledge is not power for patients: A systematic review and thematic synthesis of patient- reported barriers and facilitators to shared decision making. Patient Educ. Couns..

[46] Légaré F., Witteman H.O. (2013). Shared decision making: Examining key elements and barriers to adoption into routine clinical practice. Health Aff..

[47] Edwards M., Davies M., Edwards A. (2009). What are the external influences on information exchange and shared decision-making in healthcare consultations: A meta-synthesis of the literature. Patient Educ. Couns..

[48] Waddell A., Lennox A., Spassova G., Bragge P. (2021). Barriers and facilitators to shared decision-making in hospitals from policy to practice: A systematic review. Implement. Sci..

[49] Tringale M., Stephen G., Boylan A.M., Heneghan C. (2022). Integrating patient values and preferences in healthcare: A systematic review of qualitative evidence. BMJ Open.

